# Advances Research in Traumatic Encephalopathy

**DOI:** 10.3390/biomedicines10092287

**Published:** 2022-09-15

**Authors:** Sai Sriram, Brandon Lucke-Wold

**Affiliations:** Department of Neurosurgery, University of Florida, McKnight Brain Institute Room L2-100, 1149 South Newell Drive, Gainesville, FL 32611, USA

## 1. CTE Diagnosis

Though there are an abundance of chronic traumatic encephalopathy (CTE) cases worldwide [1], a formal diagnosis cannot be established until a histopathological analysis is conducted upon an autopsy. The paucity of reliable antemortem diagnostic prognostication tools necessitates further inquiry into recent advances in CTE diagnosis, which are detailed in the two works presented in this special edition.

The first of these articles provides a review of the latest clinical, imaging, and biomarker tools available for antemortem CTE classification [2]. This work details the diverse clinical presentations of CTE, ranging from behavioral aggression or impulsivity to cognitive memory, attention, or language decline to mood disorders of depression, mania, or anxiety [2,3,4]. Nevertheless, a purely clinical diagnostic criterion of CTE remains a controversial and unlikely prospect. The radiologic features of CTE, also covered in this review, represent a more promising diagnostic tool. Diffusion tensor imaging (DTI), an MRI technique that is able to establish white matter tract directions, has proven useful in CTE diagnosis [5], as has FDDNP, which is a radiotracer for neurofibrillary tangle (NFT) used in a PET scan [6,7]. Notably, both DTI and FDDNP-PET imaging have also been used to analyze white matter disruption in other neurodegenerative disorders such as Alzheimer’s disease (AD) [6,8]. The biomarkers t-tau, TREM2 (an inflammatory marker), and CCL11 (an eosinophil-attracting chemokine [9]) were also highlighted as potential markers for CTE in this review [2]. However, a diagnosis via biomarker remains controversial due to the poorly understood relationship between post-concussive syndrome (PCS), CTE, and AD [10]. Taken together, these findings suggest a future multifaceted approach to the antemortem diagnosis of CTE depending on a combination of clinical, radiological, and biomarker-related findings.

**Figure 1:**
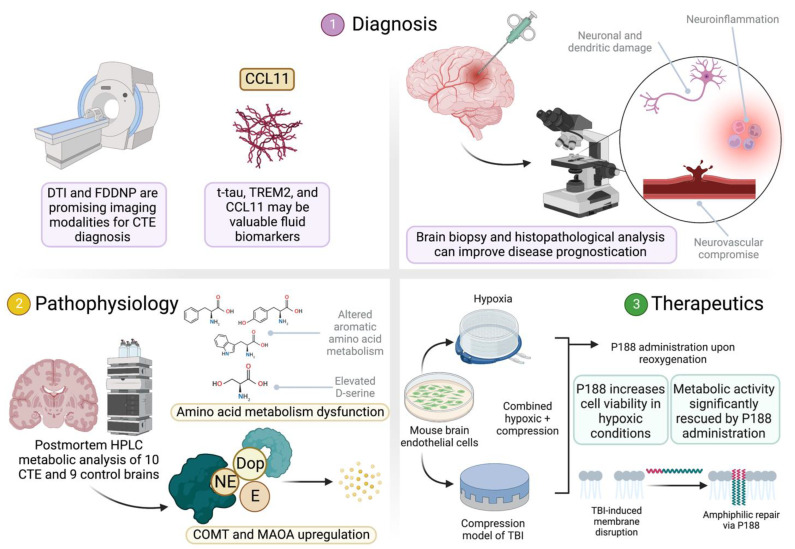
Overview of studies and findings in this special edition. Figure created with BioRender.com (accessed on 9 August 2022).

Immunohistochemical findings upon autopsy include the same proinflammatory biomarkers [11,12], blood–brain barrier (BBB) disruption [13], and neuronal loss that are characteristic to so many neurodegenerative diseases, complicating even a postmortem diagnosis of CTE. The unique pathognomonic lesion in CTE, however, is hyperphosphorylated tau (p-tau) derived neurofibrillary tangle (NFT) accumulation [14] and p-tau pathology in astrocytes [15] situated around sulcal blood vessels [12,16]. Importantly, one supportive histopathological feature of CTE is the distribution of NFTs mostly in the superficial cortical layers, as their presence in deeper cortical layers points to a diagnosis of AD [17]. A brain biopsy is not typically used for early traumatic brain injury (TBI) diagnosis due in part to its impracticability but also the difficulty in classifying histological specimens in a manner that accurately establishes disease prognosis. Despite this, one recent study included in this special edition detailed a novel grading system based on an immediate post-injury single core brain biopsy, which is predictive of the extent of neurological recovery at 6 months [18]. In this study, superior frontal gyrus biopsies were collected from 25 adult patients at an average of 14 h following injury. The histopathological evaluation of these biopsies—involving the evaluation of neuronal injury, dendritic injury, neuroinflammation, neurovascular staining, the extent of vacuolization, and the cellular appearance—was used to establish a novel injury grading system. The 6-month Glasgow outcome scale-extended (GOSE) scores were used to evaluate post-injury patient outcomes. Importantly, the injury grading system (a higher score implying worse injury) established in this study was significantly negatively correlated with 6-month GOSE scores. These results reveal the important implications this study has on the prognostication of TBI in the immediate post-injury period [18] (Figure 1).

## 2. Pathophysiology

Though p-tau accumulation in the sulcal vasculature has been implicated as the pathognomonic lesion defining CTE, the entire disease process is not fully understood. Neuroinflammation has been linked to CTE through the observation that football players with more repetitive head injuries have a greater upregulation of the inflammatory marker CD68 [11]. The pro-death effect of this inflammation is inflated by subsequent BBB disruption [19,20] and elevated oxidative stress [21]. Despite these advances in our understanding of CTE progression, inflammation, BBB damage, and free radical toxicity are also found to be elevated in AD [22,23,24], which obscures a pathophysiological mechanism specific to CTE. Clearly, the pathophysiologic differences between CTE and other neurodegenerative diseases such as AD are not well appreciated. To this end, one study in this special edition seeks to uncover unique metabolic pathways in the pathophysiology of CTE [25]. In this study, samples from the temporal lobes of 10 CTE 9 normal postmortem human brains were analyzed using three methods: (1) separation and quantification of metabolites with high-performance liquid chromatography (HPLC), (2) RNA sequencing, and (3) the direct immunohistochemistry of the samples. Following HPLC, several metabolites were found to be significantly different in level between the CTE patients and the controls, and an analysis was performed to isolate the dysregulated metabolic pathways in CTE.

One important finding in this work was the alteration in aromatic amino acid (phenylalanine, tyrosine, and tryptophan) metabolism [25]. Following previous findings revealing that aromatic amino acids may play a neuroprotective role in reducing intracranial pressure following injury [26], their dysregulated metabolism in CTE may present a new mechanism of CTE progression. D-serine was also found to be elevated, consistent with a previously reported pathologic process associated with excessive astrocytic D-serine release and resultant synaptic toxicity [27]. Interestingly, though reductions in COMT and MAOA activity are associated with behavioral aggression very similar to that seen in CTE patients [28,29], the RNA expression of the catecholamine degradation enzymes COMT and MAOA were elevated in this study. However, elevations in this metabolic pathway may contribute to p-tau accumulation and disease progression [25]. Taken together, these results shed light on the pathologic alterations to metabolic pathways in CTE.

## 3. Emerging Therapeutics

The series of events that follow TBI lead to substantial neurodegenerative changes. Importantly, these secondary changes—neuroinflammation, BBB dysfunction, and oxidative stress—are intricately related, with one leading to another in a vicious cycle culminating in neuronal death. Neurovascular and BBB dysfunction, for example, can lead to an elevated immune response as leukocytes more readily access brain tissue and promote an oxidative environment while inflammatory markers upregulate ICAMs to attract more leukocytes [30]. Theoretically, targeting any point in this cycle has the potential to diminish the cascade of subsequent events and reduce the severity of the long-term effects of TBI and has been a focus of current therapeutic research. Treatment is complicated by reperfusion injury, where reactive oxygen species induce oxidative stress following the restoration of blood flow [31]. One study in this special edition seeks to target BBB disruption with poloxamer 188 (P188), which is thought to protect endothelial cells and improve membrane defects [32]. With an amphiphilic molecular structure, this polymer has long been studied in the context of membrane repair [33]. In this study, mouse endothelial cell cultures were subjected to either a compression model of TBI, hypoxic conditions, or both, with P188 being administered upon reoxygenation. In the assessments of viability, higher concentrations of P188 were shown to improve cell survival in hypoxic conditions but not in combined hypoxic and compression conditions. Of note, this protective effect was not observed with polyethylene glycol—a compound with similar osmotic properties—treatment, isolating the treatment mechanism to the amphiphilic properties of P188. Interestingly, lactate dehydrogenase activity, a marker of tissue injury, only differed between treatment groups of P188 in those endothelial cells exposed to both hypoxia and compression. Metabolic activity, though, was significantly rescued via P188 treatment in compressive, hypoxic, or combined conditions [32]. Taken together, these promising results of endothelial cell protection warrant further investigation into the merit of P188 in vivo animal studies.

## 4. Conclusions

The works of this special edition cover a broad spectrum of topics surrounding traumatic encephalopathy and traumatic brain injury. Ultimately, antemortem CTE diagnosis is still poorly understood, and future work towards more comprehensively understanding the clinical, radiological, and biomarker-related findings are key to improving disease prognostication. A brain biopsy is a promising tool to predict 6-month cognitive outcomes in TBI but remains limited in its use and indications for the procedure. Though the pathophysiology of CTE is not well understood, we are beginning to better understand the metabolic derangements that occur as this disease process ensues. Finally, the therapies targeting the secondary neurodegenerative changes induced by TBI show promise for neuroprotection. Together, these findings paint a vivid picture for the continuation of CTE discoveries in the future.
